# Day-to-Day Glycemic Variability Using Continuous Glucose Monitors in Endurance Athletes

**DOI:** 10.1177/19322968241250355

**Published:** 2024-05-10

**Authors:** Amy-Lee M. Bowler, Louise M. Burke, Vernon G. Coffey, Gregory R. Cox

**Affiliations:** 1 Faculty of Health Sciences & Medicine, Bond University, Robina, QLD, Australia; 2 Mary Mackillop Institute for Health Research, Australian Catholic University, Watson, VIC, Australia

**Keywords:** athletes, continuous glucose monitoring, glycemic variability, interstitial glucose

## Abstract

**Objectives:**

The application of continuous glucose monitors (CGMs) to measure interstitial glucose in athletic populations is limited by the lack of accepted athlete-specific reference values. The aim of this study was to develop athlete-specific reference ranges for glycemic variability under standardized diet and exercise conditions.

**Methods:**

A total of 12 elite racewalkers (n = 7 men, 22.4 ± 3.5 years, VO_2max_ 61.6 ± 7.3 mL kg^−1^ min^−1^) completed two 4-d trials separated by 4-d. Athletes were provided a high-energy, high-carbohydrate diet (225 ± 1.6 kJ kg^−1^ day^−1^, 8.4 ± 0.3 g kg^−1^ day^−1^ carbohydrate) and completed standardized daily exercise. The timing of food consumed and exercise undertaken were matched each day across the 4-d trials. Interstitial glucose data were collected via Freestyle Libre 2 CGMs. Glycemic variability was calculated as the mean amplitude of glycemic excursions (MAGEs), mean of daily differences (MODD), and standard deviation (SD).

**Results:**

Twenty-four hour MODD, MAGE, and SD for interstitial glucose were 12.6 ± 1.8 mg/dL (0.7 ± 0.1 mmol/L), 36.0 ± 5.4 mg/dL (2.0 ± 0.3 mmol/L), and 16.2 ± 1.8 mg/dL (0.9 ± 0.1 mmol/L), respectively. Twenty-four hour mean glucose (MG; 102.6 ± 5.4 mg/dL [5.7 ± 0.3 mmol/L]) was higher than overnight (91.8 ± 5.4 mg/dL [5.1 ± 0.3 mmol/L]; *P* < .0001) and was lower in women than men (99.0 ± 3.6 mg/dL [5.5 ± 0.2 mmol/L] vs 104.4 ± 3.6 mg/dL [5.8 ± 0.2 mmol/L]; *P* = .059, d = 1.4).

**Conclusions:**

This study provides reference indices under standardized diet and exercise conditions for glycemic variability derived from CGMs in endurance athletes which are similar than previously reported for healthy individuals, despite strenuous daily training and a high daily energy and carbohydrate diet.

## Introduction

Continuous glucose monitors (CGMs) are devices that capture daily glucose dynamics, providing the user with (near) real-time measures of interstitial glucose concentration.^1^ To collect continuous glucose metrics, CGMs are implanted into the subcutaneous tissue of a site approved for use by the manufacturer, typically the back of the upper arm, the lower back or the abdomen.^2,3^ The implanted sensor then uses a glucose-oxidase (GOx) reaction to generate a current, analogous to the glucose concentration within the interstitial fluid, which is subsequently used to estimate blood glucose concentration.^4^ Once the device is implanted, information is transmitted via wireless technology to either a data receiver or mobile smartphone which enables the user to view glucose snapshots every 1 to 15 minutes alongside 24 hour periods of continuous glucose data.

Historically, these devices have been used by individuals living with diabetes to facilitate prompt management of undesirable glucose fluctuations. Here, CGM capture of time series data reflecting blood glucose responses to recent food and fluid intake and physical activity allows the adjustment of dietary intake and/or insulin dosing in (near) real-time.^1^ Indeed, it has been shown that the glucose estimates provided by CGMs are comparable to blood glucose data collected via traditional methods, such as venous blood sampling, typically used in hospital settings or laboratories.^5^

Following the success of these devices in the management of diabetes mellitus, CGMs have been considered as a tool to inform daily training and fueling practices in healthy, active individuals and athletes.^6^ Recently, athlete-specific devices such as the Abbott Libre Sense Glucose Sport Biosensor (Abbott Diabetes Care, Chicago, Illinois) and software platforms (ie, Supersapiens, Ultrahuman) have been designed with the aim of providing active individuals and athletes with access to real-time glucose metrics including 24 hour glucose averages, daily glucose patterns, and hypoglycemic episodes. Although previous studies have established reference ranges for glycemic variability in individuals with diabetes mellitus^7^ and healthy non-athletic populations,^8^ there are currently no accepted reference values for athletes under standardized diet and exercise conditions. To support the interpretation of CGM-derived glucose values in future diet and exercise interventions, accepted athlete-specific reference ranges need to be established. Hence, the aim of this study was to develop athlete-specific CGM-derived glycemic variability markers under standardized exercise and dietary protocols among endurance athletes.

## Methods

### Subjects

A total of 12 elite racewalkers (men n = 7, 22.5 ± 3.5 years, 60.1 ± 6.3 kg, VO_2max_ 61.6 ± 7.3 mL kg^−1^ min^−1^) attending a training camp at the Australian Institute of Sport (AIS) completed the study. Two of the 12 participants were classified as Tier 5: World Class athletes, while three were Tier 4: Elite/International Level and five were Tier 3: Highly Trained/National Level.^9^ The research procedures were approved by the relevant ethics committee in Australia (BUHREC; no. AB03648), and participants provided written informed consent prior to study commencement.

### Standardized Dietary and Exercise Protocol

**Figure 1.**
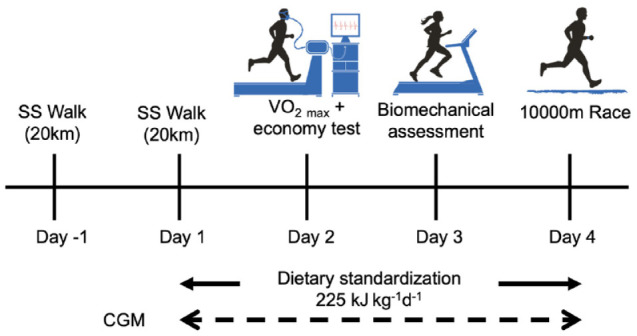
Study design schematic. Participants (n = 12) completed two 4-d trials across consecutive weeks. SS: steady state. CGM: continuous glucose monitor.

The study was implemented during a four week training camp comprising two 4-d trial periods, each separated by three days (Figure 1). Athletes resided onsite in athlete accommodation for the duration of the study, allowing for control and monitoring of training, food and fluid intake, and sleep. During each 4-d trial period, athletes adhered to a weight maintaining standardized diet consisting of energy 225 kJ kg^−1^ day^−1^, carbohydrate 8.5 g kg^−1^ day^−1^, protein 2.1 g kg^−1^ day^−1^. All meals and snacks were produced according to standardized recipes and weighed for subsequent nutrient analysis (Nutritics Ltd, Dublin, Ireland) by a qualified sports dietitian. Main meals were provided at fixed time points in the AIS Dining Hall (0800-0830, 1230-1300, and 1900-1930). Participants were instructed to consume only the meals and snacks provided. Standardized exercise protocols were followed throughout each 4-d trial period and included a steady-state (SS) race-walking bout on day 1, economy and biomechanical testing and resistance training on days 2 and 3, respectively, and a 10 000 m race walk on day 4. All SS race-walking bouts were undertaken on the same road training circuit matched across the two 4-d trials. Standardized race-walking sessions were completed between the two 4-d trials, including the day before each trial commenced.

### Determination of Interstitial Glucose Concentrations

A CGM sensor (Abbott Freestyle Libre 2, Abbott Diabetes Care) was inserted into the back of the upper arm of each participant ~ 24 hours before each 4-d trial to allow 24 hours for calibration prior to data collection. CGMs were removed and subsequently replaced following the first 4-d trial. The CGM device used in this study collected interstitial glucose values every minute and was stored and subsequently reported every 15 minutes. Glucose data were downloaded, and glucose profiles evaluated for glycemic variability from the LibreView application (Abbott Diabetes Care).

### Statistical Analysis

Glycemic variability was assessed by calculating the mean amplitude of glycemic excursion (MAGE), mean of daily differences (MODD), mean glucose (MG), standard deviation (SD) and percent time spent in hypo-, normo-, and hyperglycemia using the *cgmanalysis* package for R Studio (Version 2022.02.1, R Studio PBC, Boston, Massachusetts). MODD was calculated as the mean of differences between glucose values on energy-matched days at the same time across trials (eg, Trial 1 Day 1 at 0800 vs Trial 2 Day 1 at 0800). MAGE was determined by calculating the arithmetic mean of the differences > 1SD between peaks and nadirs during each specified period across all eight days. Individual MG, SD, and time in range values were calculated as an average across all eight days. Measures of glycemic variability (MODD, MAGE, MG, SD) were calculated for 24 hours, daytime (06:00-22:00) and overnight (22:00-06:00) time periods. Where appropriate, data are expressed as the mean ± SD or median (interquartile range). To compare differences in glycemic variability markers at various time points and between sexes, one-way analyses of variance (ANOVAs) or *t*-tests were employed. The Pearson correlation was used to assess relationships between indices of glycemic variability. Effect sizes were calculated using Cohen’s d with thresholds for small (0.2), moderate (0.5), and large (0.8) interpreted according to Cohen.^10^ Statistical analyses were conducted using GraphPad Prism (Version 9.1.2, GraphPad Software Inc, La Jolla, California) and effect sizes were calculated using Microsoft Excel (Microsoft Corporation, Redmond, Washington). Statistical significance was set at *P* < .05.

## Results

Energy intake during the two 4-d trial periods was 224.6 ± 1.6 kJ kg^−1^ d^−1^, carbohydrate 8.4 ± 0.3 g kg^−1^ d^−1^, protein 2.1 g kg^−1^ d^−1^, and fat 1.2 g kg^−1^ d^−1^. There was no difference in daily energy (Δ 47.7 ± 115.7 kJ kg^−1^ d^−1^; *P* = .44) or macronutrient intake (carbohydrate Δ 2.1 ± 3.9 g kg^−1^ d^−1^, protein Δ 0.9 ± 1.4 g kg^−1^ d^−1^, fat Δ 0.6 ± 0.7 g kg^−1^ d^−1^; *P* > .84) between the two 4-d trial periods or between men and women participants (Δ 0.05 ± 1.7 kJ kg^−1^d^−1^; *P* = .98).

**Figure 2.**
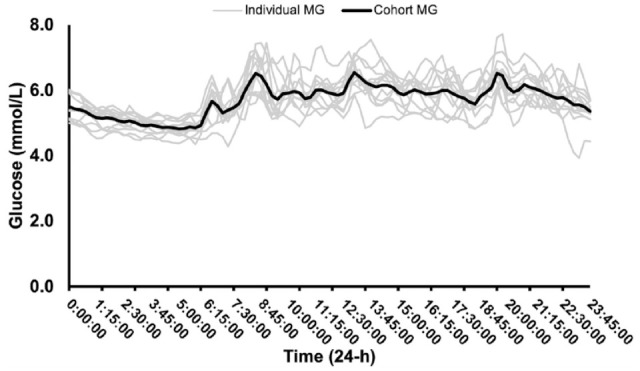
Individual and cohort mean 24 hour glucose (MG) over eight days of continuous glucose monitoring (n = 12).

Twelve CGM data sets providing a total of 168.0 ± 25.1 hours of data were collected across the two 4-d trial periods (Figure 2). Due to delayed synching and/or accidental sensor removal, up to 25 hours of data was lost across the two 4-d trial periods (192 hours total) for each participant. Glucose values trended downward overnight with the lowest glucose values early morning (91.8 mg/dL [5.1 mmol/L] at 04:15), following an overnight fast (~8-9 h). Glucose values rose throughout the day and peaked(115.2 mg/dL [6.4 mmol/L], 115.2 mg/dL [6.4 mmol/L], and 117.0 mg/dL [6.5 mmol/L)], following the ingestion of main meals: breakfast (08:00-08:30, energy 45.2 ± 3.7 kJ kg^−1^ d^−1^, carbohydrate 1.4 ± 0.2 g kg^−1^ d^−1^), lunch (12:30-13:00, energy 47.7 ± 3.1 kJ kg^−1^ d^−1^, carbohydrate, 1.5 ± 0.2 g kg^−1^ d^−1^), and dinner (19:00-19:30, energy 45.6 ± 2.9 kJ kg^−1^ d^−1^, carbohydrate 1.6 ± 0.2 g kg^−1^ d^−1^), respectively.

Mean of daily differences, calculated as the MODD between the two 4-d periods, was 12.6 ± 1.8 mg/dL (0.7 ± 0.1 mmol/L). There were no differences in MG, MAGE, or SD (*P* > .3) between the two 4-d trial periods (Table 1). As such, data for these variables are reported across the 8-d of the study. Twenty-four hour MG was 102.6 ± 5.4 mg/dL (5.7 ± 0.3 mmol/L) and was significantly higher than overnight 91.8 ± 5.4 mg/dL (5.1 ± 0.3 mmol/L; *P* = .0001, d = 2.0). MAGE was 36.0 ± 5.4 mg/dL (2.0 ± 0.3 mmol/L), whereas SD was 16.2 ± 1.8 mg/dL (0.9 ± 0.1 mmol/L). MAGE, MODD, and SD were normally distributed among participants. The 95th percentile values were 46.8 mg/dL (2.6 mmol/L; MAGE), 16.2 mg/dL (0.9 mmol/L; MODD), and 19.8 mg/dL (1.1 mmol/L; SD). There was a significant difference between overnight and 24 hour MG, MAGE, MODD, and SD (*P* < .05; Table 1). The Pearson correlation analysis indicated a significant positive correlation between MAGE and SD (r = 0.81, *P* < .005).

**Table 1.** Comparison of CGM-Derived Measures of Glycemic Variability for Participants Across 24 hours, Daytime (06:00-22:00 h) and Overnight (22:00-06:00 h). Where Appropriate, Data Are Presented as Mean ± SD (n = 12).

		24 h	Daytime (06:00-22:00 h)	Overnight (22:00-06:00 h)
MG	mg dL^−1^ (mmol L^−1^)	102.6 ± 5.4 (5.7 ± 0.3)	108 ± 3.6 (6.0 ± 0.2)*^#^	91.8 ± 5.4 (5.1 ± 0.3)*
MODD	mg dL^−1^ (mmol L^−1^)	12.6 ± 1.8 (0.7 ± 0.1)	16.2 ± 1.8 (0.9 ± 0.1)*^#^	9.0 ± 1.8 (0.5 ± 0.1)*
MAGE	mg dL^−1^ (mmol L^−1^)	36.0 ± 5.4 (2.0 ± 0.3)	39.6 ± 5.4 (2.2 ± 0.3)^#^	25.2 ± 7.2 (1.4 ± 0.4)*
SD	mg dL^−1^ (mmol L^−1^)	16.2 ± 1.8 (0.9 ± 0.1)	18.0 ± 1.8 (1.0 ± 0.1)^#^	10.8 ± 3.6 (0.6 ± 0.2)*
AUC	mg dL^−1^ (mmol L^−1^ h^−1^)	6093 ± 280.8 (338.5 ± 15.6)	6396.2 ± 270.0 (355.4 ± 15.0)*^#^	5581.8 ± 268.2 (310.1 ± 14.9)*
% CV		0.2 ± 0.0	0.2 ± 0.0	0.1 ± 0.0
Hypoglycemia >72.0 mg/dL	%	0.5 (7.3)	0.0 (3.0)	0.8 (22.5)
Euglycemia 72.0-144.0 mg/dL	%	96.3 ± 2.4	96.0 ± 2.4	99.3 (22.5)
Hyperglycemia >144.0 mg/dL	%	2.4 ± 1.6	3.5 ± 2.4^#^	0.0*

CGM: continuous glucose monitor; MG: mean glucose; MODD: mean of daily differences; MAGE: mean amplitude of glycemic excursion; SD: standard deviation; %CV: percentage coefficient of variation for glucose.

* Denotes significant difference from 24 hours (*P* < .02).

# Denotes significant difference from overnight (*P* < .0001).

**Figure 3.**
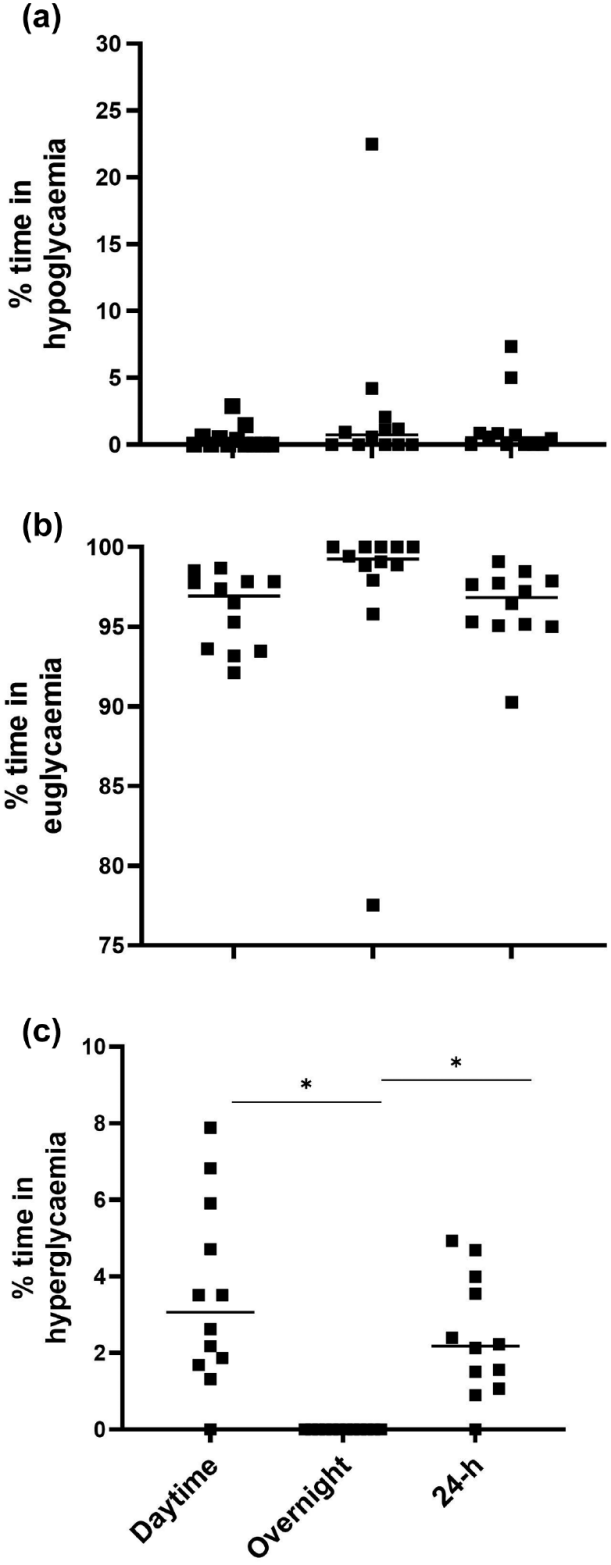
Percent time spent in either (a) hypoglycemia (<72.0 mg/dL [< 4.0 mmol/L]), (b) euglycemia (72.0-144.0 mg/dL [4.0-8.0. mmol/L]), or (c) hyperglycemia (>144.0 mg/dL [> 8.0 mmol/L]) across all 8-d of the trial. *Denotes a significant difference (*P* < .0030).

Across all 8-d of the study, 96.3 ± 2.4% of time was spent in euglycemia (72.0-144.0 mg/dL [4.0-8.0 mmol/L]), 1.3 ± 2.4% in hypoglycemia (< 72.0 mg/dL [<4.0 mmol/L]), and 2.4 ± 1.6% in hyperglycemia (> 144.0 mg/dL [> 8.0 mmol/L]). There were no differences in percent time spent in euglycemia between daytime (06:00-22:00), overnight (22:00-06:00), and 24 hour periods (96.0 ± 2.4%, 97.3 ± 6.3% and 96.3 ± 2.4%, respectively; Figure 3). However, no time was spent in hyperglycemia during overnight hours which was significantly lower than 24 hours (2.4 ± 1.6%; *P* < .0001) and daytime hours (3.5 ± 2.4%; *P* < .0001, d = 0.5; Figure 3). There was a significant difference between 24 hour (68.4 ± 9.0 mg/dL [3.8 ± 0.5 mmol/L] to 180.0 ± 2.3 mg/dL [10.0 ± 1.3] mmol/L]) and overnight glucose range (70.2 ± 9.0 mg/dL [3.9 ± 0.5 mmol/L] to 126.0 ± 12.6 mg/dL [7.0 ± 0.7 mmol/L]); *P* < .0001.

**Figure 4.**
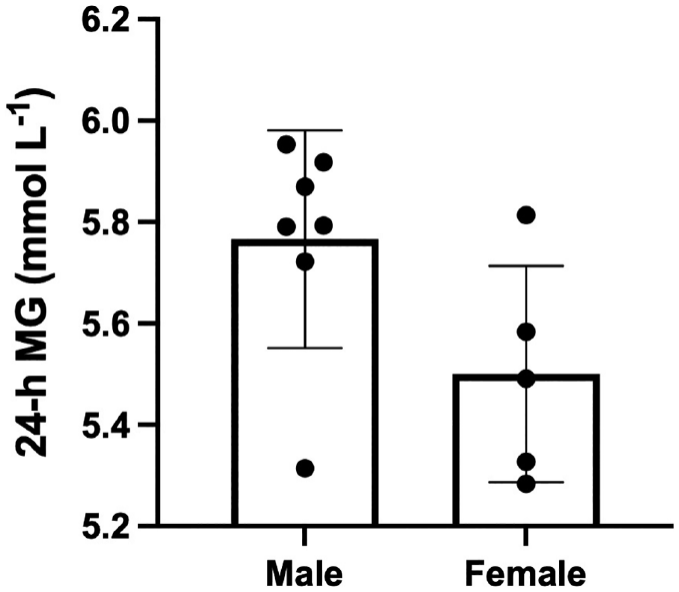
Twenty-four hour MG in men (n = 7) vs women (n = 5) across all 8-d of the trial. Reported as mean ± SD. MG: mean glucose.

No significant differences in MAGE, MODD, or SD were observed with age. Neither MAGE, SD, nor MG were different between men and women; however, there was a strong effect that approached significance for 24 hour MG between men (104.4 ± 3.6 mg/dL [5.8 ± 0.2 mmol/L]) and women (99.0 ± 3.6 mg/dL [5.5 ± 0.2 mmol/L]; *P* = .06, d = 1.4; Figure 4).

Irrespective of trial, pre-race MG on day 4 was significantly lower (93.6 ± 14.4 mg/dL [5.2 ± 0.8 mmol/L]) compared with during race MG (106.2 ± 14.4 mg/dL [5.9 ± 0.8, *P* = .02, d = 0.9]) and after race MG (111.6 ± 10.8 mg/dL [6.2 ± 0.6 mmol/L, *P* = .002, d = 0.4]). There was no difference between MG during and after the race (*P* = .50).

## Discussion

This is the first study to report CGM-derived 24 hour, daytime (06:00-22:00), and overnight (22:00-06:00) glucose variability measures for elite endurance athletes under standardized exercise and dietary conditions. The findings were three-fold. First, glycemic variability in elite endurance athletes was similar to previously reported observations of healthy individuals, yet lower than those living with diabetes, despite consuming a high-energy, high-carbohydrate diet while undertaking high daily training loads. Second, participants incurred lower glucose variability overnight compared with 24 hour and daytime measures. And third, despite standardized diet and exercise conditions adjusted for individual differences in body mass, men tended to have higher 24 hour MG compared with women.

To characterize the day-to-day variation of CGM-measured glycemia, parameters of glycemic variability (MODD, MAGE, and SD) were used to measure both inter-day (MODD) and intra-day (MAGE, SD) fluctuations in interstitial glucose. MODD characterizes day-to-day glucose variability under standardized conditions, whereas MAGE best quantifies the average magnitude of glucose fluctuations within a 24 hour period. These complementary measures, along with 24 hour MG, provide the best estimate of an individual’s whole-body glycemic stability across a given period.^11^ This study revealed that MODD was 12.6 ± 1.8 mg/dL (0.7 ± 0.1 mmol/L). MODD is an accepted indicator of inter-day glycemic variability and was calculated as the MODD between the two 4-d standardized diet and exercise periods.^12^ It is unsurprising that MODD was low given the timing and structure of exercise sessions as well as the timing and composition of meals and snacks were replicated between the two 4-d periods. In contrast, high MODD values have previously been shown to reflect irregular lifestyle patterns.^13^ MAGE and SD averaged across 8-d of the study were 36.0 ± 5.4 mg/dL (2.0 ± 0.3 mmol/L) and 16.2 ± 1.8 mg/dL (0.9 ± 0.1), respectively. A previous study reported similar values for MODD (18.0 ± 3.6 mg/dL [1.0 ± 0.2 mmol/L]), MAGE(30.6 ± 9.0 mg/dL [1.7 ± 0.5 mmol/L ]), and SD (14.4 ± 5.4 mg/dL [0.8 ± 0.3 mmol/L]) in healthy sedentary (n = 29) individuals without diabetes under 3-d of standardized diet and exercise conditions.^8^ Notably, these results are markedly lower than those reported in individuals living with type 2 diabetes mellitus adhering to a 3-d standardized diet (MAGE 104.4 ± 34.2 mg/dL [5.8 ± 1.9 mmol/L], MODD 27.0 ± 10.8 mg/dL [1.5 ± 0.6 mmol/L]).^14^ Indeed, individuals with type 2 diabetes mellitus display metabolic impairments associated with insulin resistance and lack the cellular machinery to readily switch fuel utilization between glucose and fatty acids. This lack of metabolic flexibility in glucose and fatty acid oxidation during the post-absorptive period results in greater daily glycemic variability.^15^

Despite a high-energy and carbohydrate diet, elite racewalkers demonstrated similar glycemic control in comparison to previously reported values for healthy, non-active individuals.^8^ In contrast to our findings, subelite and elite athletes have been reported to have greater glycemic variability in free living conditions.^16-18^ Without detailed accounts of dietary intakes and daily training in previous studies, it is difficult to explain whether the differences observed are in response to adequacy of daily fueling^16^ or mitochondrial dysfunction-associated excessive training.^17^ In this study, a standardized diet with an identical energy and carbohydrate content was consumed each day rather than fluctuating according to the daily training load. This was replicated across the two 4-d trials. Given energy intakes provided to participants were relative to individual body mass, were evenly distributed across each main meal and snack, and consumed in a standardized schedule throughout the day, it is unsurprising that we observed better glucose control and reductions in overall glycemic variability compared with situations where athletes are free living with unstructured dietary intakes. In contrast, one participant in this study spent considerable time (~ 23%) in hypoglycemia (Figure 3). It is unlikely this participant was non-compliant to diet and exercise standardization as all participants were closely monitored throughout the study. However, the athlete in question followed a vegetarian diet and was provided with higher fiber protein containing foods such as chickpeas, lentils, and beans to meet prescribed dietary intakes which may reduce energy absorption,^19^ subsequently decreasing mean interstitial glucose. Whether a daily eating plan in which energy and macronutrient intakes were more closely aligned to changes in an athlete’s training schedule would result in further improved glycemic variability (measured with CGMs) is currently unknown. This should be the focus of future research as it may provide an opportunity to make real-time alterations to daily fueling strategies amongst athletes.

Endurance training achieves favorable metabolic adaptations that promote lipid oxidation and decrease sympathetic nervous system activity.^20^ Exercise intensity primarily determines relative contribution of carbohydrate and lipid to energy production during exercise among athletic individuals, with dietary intake, training status, and sex subsequently influencing substrate selection.^21^ Together, these adaptations to endurance exercise enhance metabolic flexibility and allow athletes to respond to changes in exogenous substrate availability to meet the energy requirements associated with exercise.^15^ Participants in this study were provided a high-carbohydrate diet which maintained MG within normoglycemic range before, during and after (5.2-6.2 mmol/L [93.6-111.6 mg/dL]) a 10 km race walk completed on day 4 of each trial. Furthermore, racewalkers typically exhibit a relatively ectomorphic somatotype which has been correlated to decreased blood glucose fluctuations.^22^ It is understood that individuals who display endomorphic characteristics exhibit higher blood glucose concentrations, whereas no relationship has been reported for mesomorphic attributes.^22,23^ These findings may have implications for power- and/or team-based sport athletes where body composition varies depending on the athlete’s event and/or position.^24^ Given that somatotype may influence glucose variability or the technical error of measurement, it is suggested that non-endurance athletes may respond differently under the same standardized dietary and training conditions undertaken in this study. As such, further investigation is warranted to establish glycemic variability ranges for power, strength, and team sport athletes.

A key finding from this study is that all measures of glycemic variability (ie, MG, MAGE, MODD and SD) were lower during overnight hours (22:00-06:00), compared with daytime hours (06:00-22:00), with athletes spending less time in hyperglycemia overnight (2.4 ± 1.6% vs 0.0 ± 0.0%, respectively). The timing, type, and quantity of dietary carbohydrate ingested before, during, and after exercise and at meals and snacks throughout the day, alongside the macronutrient (carbohydrate, protein, and fat) composition of the diet will influence glucose homeostasis and measures of glycemic variability.^25^ Furthermore, the inherent intricacies associated with the balance between feed-forward and feedback mechanisms such as the exponential increases in norepinephrine and epinephrine that occur with gradations in exercise intensity also effect glucose control throughout the day.^26^ Indeed, episodes of hyperglycemia have been reported in response to intense exercise training in men due to higher levels of epinephrine resulting in augmented hepatic glycogenolysis.^27^ In contrast, the lowest overnight blood glucose levels reflect the metabolic systems’ ability to return to basal glucose concentration with the removal of the interaction of diet and exercise (activity).^16^ As the overnight period is less likely influenced by factors that increase glycemic variability, it is unsurprising that this study observed a decrease in CGM-derived measures of glycemic variability overnight compared with 24 hours and daytime time periods. We have previously suggested that CGM data may be indicative of overall energy status, particularly when a focus is placed on the overnight period where the acute interactions between diet and exercise are removed.^6^ Thus, analyzing this period more closely for perturbations to glycemic control may prove useful, particularly in athletic populations where daily energy requirements fluctuate considerably in response to variations in daily exercise patterns. Athletes are required to align their dietary intake on a daily basis to these fluctuations to avoid a significant mismatch with daily energy needs. To date, most observations of CGM-derived overnight glucose stability have been focused on individuals with diabetes,^28,29^ with the exception of one study reporting elevated overnight CGM-derived glucose responses in subelite endurance athletes undertaking a maximal exercise test.^30^

Despite no significant differences in energy or macronutrient intake relative to body mass, 24 hour MG was higher in men compared with women in this study. A possible explanation for this disparity could be lower circulating blood glucose during and immediately following sustained moderate intensity exercise in active women compared with men.^31,32^ Women have been reported to generate significantly lower glucagon and epinephrine responses to exercise and may have a reduced exercise glucose turnover which could explain the lower values observed in this study.^31,33^ Due to the nature of the study design, it was not possible to standardize for menstrual cycle phase among women which might be a contributing factor to the observed differences in MG between men and women. Indeed, cyclical fluctuations in glucose have been previously reported across the menstrual cycle in healthy women.^34^ Future studies should seek to explore the potential effect of hormonal fluctuations associated with menstrual cycle on CGM-derived glucose measures in athletes.

## Conclusions

This is the first study to characterize CGM-measured glycemic variability in elite endurance athletes under standardized exercise and dietary conditions. As the use of CGM technology in sport is rapidly expanding, the reference values for CGM-derived glucose variability in endurance athletes established in this study are a key tool for researchers and practitioners. Whether CGM-derived interstitial glucose values outside of those reported here reflect disturbances to physiological functioning as a result of daily training and fueling or an athlete’s response to training is yet to be determined and should be the focus of future research. Nonetheless, in light of our findings, future analysis of laboratory-based CGM data in endurance athletes can be more clearly interpreted using CGM-measured glycemic variability reference ranges established in this study.
